# Does moxonidine reduce Achilles tendon or musculoskeletal pain in women with polycystic ovarian syndrome? A secondary analysis of a randomised controlled trial

**DOI:** 10.1186/s12902-020-00610-8

**Published:** 2020-08-26

**Authors:** Jacob Jewson, Elisabeth Lambert, Carolina Sari, Eveline Jona, Soulmaz Shorakae, Gavin Lambert, Jamie Gaida

**Affiliations:** 1 Faculty of Medicine, Nursing and Health Sciences, Monash University, Melbourne, Victoria Australia; 2 Present Address: Olympic Park Sports Medicine Centre, Melbourne, Victoria Australia; 3 Iverson Health Innovation Research Institute and School of Health Sciences, Swinburne University of Technology, Melbourne, Victoria Australia; 4 Baker Heart and Diabetes Institute, Melbourne, Victoria Australia; 5 Monash Centre for Health Research and Implementation, School of Public Health and Preventive Medicine, Monash University, Melbourne, Victoria Australia; 6 Diabetes and Vascular Medicine Unit, Monash Health, Melbourne, Victoria Australia; 7 University of Canberra Research Institute for Sport and Exercise (UCRISE), Canberra, ACT Australia; 8 Discipline of Physiotherapy, University of Canberra, Canberra, ACT Australia

**Keywords:** Tendinopathy, Musculoskeletal pain, Sympatholytics, Metabolic syndrome, Sympathetic nervous system, Insulin resistance, Polycystic ovarian syndrome

## Abstract

**Background:**

Sympathetic activity and insulin resistance have recently been linked with chronic tendon and musculoskeletal pain. Polycystic ovarian syndrome is linked with insulin resistance and increased sympathetic drive and was therefore an appropriate condition to study the effects of modulating sympathetic activity on Achilles tendon and musculoskeletal symptoms.

**Methods:**

A secondary analysis of a double-blinded, randomised controlled trial on women with polycystic ovarian syndrome was conducted. Participants received 12 weeks of moxonidine (*n* = 14) or placebo (*n* = 18). Musculoskeletal symptom and Victorian Institute of Sport Assessment – Achilles (VISA-A) questionnaires were distributed, and ultrasound tissue characterisation quantified tendon structure at 0 and 12 weeks. 2-way ANOVA was used for multiple comparisons.

**Results:**

There was no difference in mean change in musculoskeletal symptoms (− 0.6 ± 1.7 vs − 0.4 ± 1.8, *p* = 0.69) or VISA-A (moxonidine − 0.2 ± 8.8 vs placebo + 4.2 ± 14.6, *p* = 0.24) attributable to the intervention. There was no difference in any measures of Achilles structure. Moxonidine did not reduce sympathetic drive when compared to placebo.

**Conclusions:**

This was the first study to investigate the effects of blocking sympathetic drive on musculoskeletal and Achilles tendon symptoms in a metabolically diverse population. While the study was limited by small sample size and lack of sympathetic modulation, moxonidine did not change tendon pain/structure or musculoskeletal symptoms.

**Trial registration:**

ClinicalTrials.gov, NCT01504321. Registered 5 January 2012.

## Background

Achilles tendinopathy is a difficult to manage musculoskeletal (MSK) condition with an incompletely understood pathophysiology [1, 2] that adversely affects quality of life [3]. While typically associated with running, this painful condition also affects up to 6% of the non-athletic general population [4]. The sympathetic nervous system (SNS), insulin resistance, type 2 diabetes mellitus (T2DM), dyslipidaemia, and visceral adiposity have been identified as potential contributors to MSK pain, including tendinopathy [5–7]. There is evidence that the SNS could underpin these metabolic features associated with MSK and tendon pain [8].

Recently, SNS function in painful tendons has been investigated in a small number of studies. A systematic review of microscopy studies has revealed increased markers of catecholamines synthesis (e.g. tyrosine hydroxylase) and adrenoreceptors in the paratendinous tissue, as well as increased adrenoreceptor like substances on abnormal tenocytes within the tendon proper, in biopsies from painful tendons [9]. In addition, upregulation of the SNS contributes to chronic pain [10]. Among individuals with tendon pain, SNS activity is higher in people with longer symptom duration and correlates with poorer tendon structure [11]. Together, these observations raise the possibility that the SNS plays a role in the chronicity of tendinopathy and structural change within the tendon.

Polycystic ovarian syndrome (PCOS) is a common clinical disorder among women that presents as a combination of hyperandrogenism, ovulatory dysfunction, and polycystic ovaries [12]. Other clinical features include insulin resistance, T2DM, infertility, and visceral obesity [13]. PCOS has also been associated with increased sympathetic activity, independent of other metabolic factors [13, 14].

Only one study has examined in vivo sympathetic drive in tendinopathy, and this was in a metabolically-normal population with clinical symptoms of tendon pain [11]. A double-blinded randomised controlled trial of moxonidine (a sympatholytic medication) to treat the symptoms of PCOS (Clinical Trial registration NCT01504321) [15] provided a unique opportunity to document the change in tendon and MSK symptoms in response to sympathetic nervous inhibition in a metabolically diverse population.

As moxonidine is a centrally acting imidazoline 1 (and to a lesser extent adrenoreceptor type-2) agonist which has been shown to reduce both sympathetic drive and insulin resistance [15], it was hypothesised that this may improve MSK and Achilles tendon pain, which both have links to increased SNS drive and metabolic dysfunction. Therefore, the aim of this paper was to examine the effects of moxonidine on Achilles and general MSK pain (primary outcome) and on Achilles tendon structure (secondary outcome) in women with a diagnosis of PCOS.

## Methods

### Study design and participants

This study is a secondary analysis of outcomes measured during a previously published double-blinded randomised controlled trial (RCT) [15]. The study was conducted in accordance with the Declaration of Helsinki and the protocol was granted ethics approval by the Alfred Hospital (in conjunction with Monash Health) Human Research Ethics Committee (approval HREC/12/Alfred/10) and amended to include the outcomes required for this portion of the study. Essentially, the MSK and Achilles data were collected alongside the parent RCT and the outcomes assessed separate to the aforementioned paper.

Participants were recruited from the Melbourne metropolitan area according to the eligibility criteria previously described [15], after written informed consent was obtained and appropriate medical examination was performed. Importantly, participants were recruited primarily based on a diagnosis of PCOS according to the parent study, and not based on any prior MSK or Achilles tendon symptoms.

**Fig. 1:**
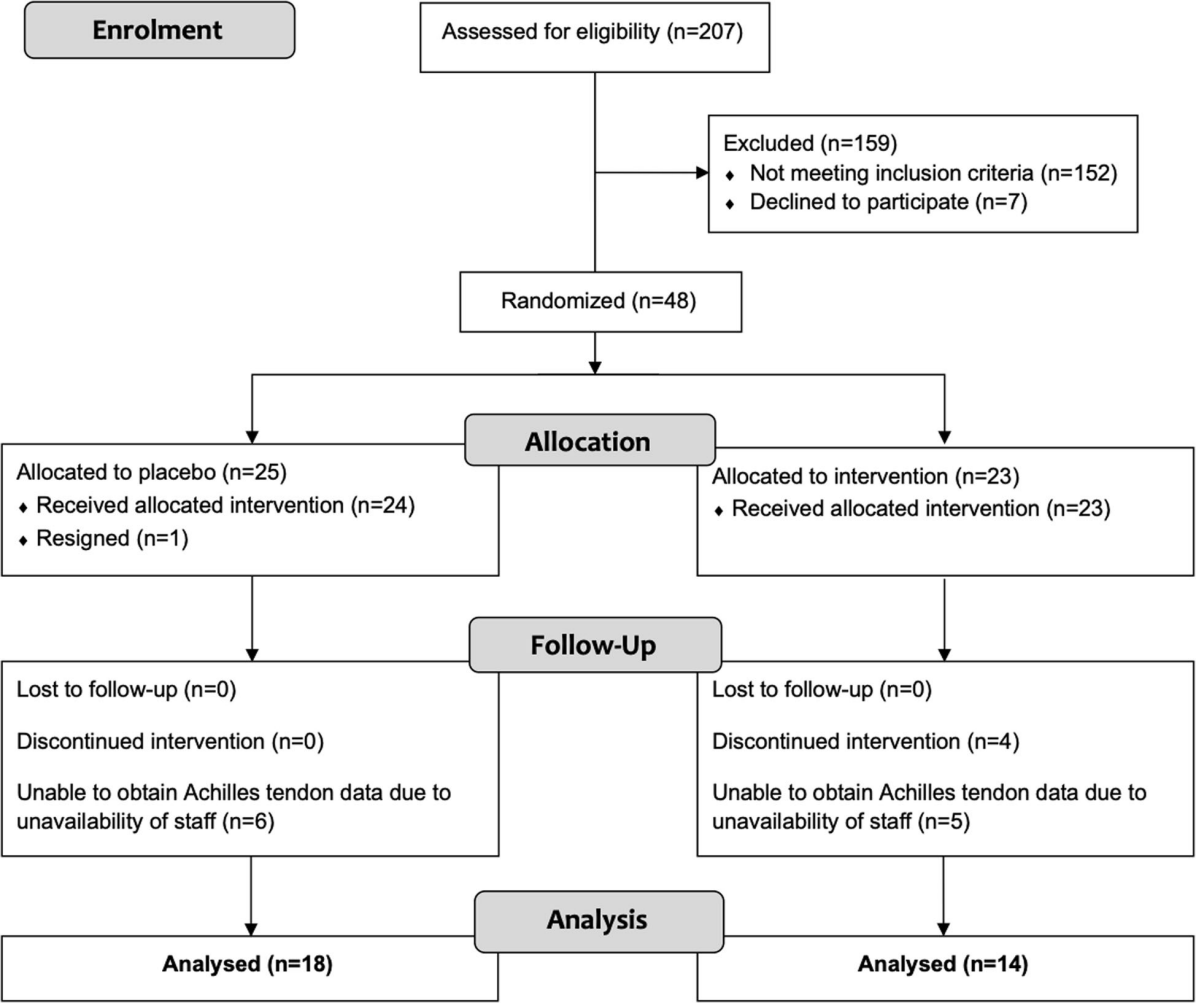
Consort flow diagram of recruitment for the study

Participants were randomised by the Alfred Hospital Clinical Trial Pharmacy to either the moxonidine or placebo between June 2013 and August 2015. However, the final patient to be included in the Achilles/MSK analysis completed the intervention in January 2015 due to availability of staff and resources (Fig. 1). This was performed using simple block randomisation with block sizes of 10 (5 moxonidine and 5 placebo) [15]. Both assessors and participants were blinded to group allocation until after analysis of results by way of a numbered code and an additional code for digital files [16].

### Intervention and follow up

Participants on hormonal contraceptives were asked to cease this medication and undergo a three-month washout period to be involved in this trial. Once eligibility for the study was confirmed by medical examination, participants had a subjective and objective Achilles assessment. Participants were then provided 12 weeks of medication (either active or placebo). Moxonidine dosing was 0.2 mg daily initially, up titrated to 0.4 mg daily in 2 weeks [15]. All participants were followed up after 12-weeks of medication treatment and all assessments were then repeated for comparison to pre-intervention data. No exercises were prescribed, and participants were advised to maintain their normal routine.

### History and assessment of metabolic parameters

Participants provided a basic medical history as previously described. Body mass index (BMI) was calculated using height and weight, while waist and hip circumference were measured to provide a ratio (WHR). Resting systolic (SBP) and diastolic (DBP) were also measured (Digital Automatic Blood Pressure Monitor HEM907, Omron Electronics Pty Ltd), with mean arterial pressure (MAP) estimated as one-third SBP plus two-thirds DBP.

All participants completed an oral glucose tolerance test. This involved drinking a 75 g glucose drink and blood collection at 0 and 120 min. Both glucose and insulin were measured at each time point to allow calculation of the Matsuda index [17], and homeostatic model assessment (HOMA) index [18] as indicators of insulin resistance.

### Musculoskeletal and Achilles tendon questionnaires

Each participant completed two questionnaires on MSK symptoms at the start and end of the trial. The first assessed general MSK and joint pains in the past month. This questionnaire directly replicated that used by Taylor et al. [19]. The questionnaire asked about pain of more than one-week duration in the previous month affecting the i) back, ii) neck, iii) shoulder, iv) elbow, v) hand, vi) hip, vii) knee, viii) foot, or ix) most joints. The final question was “I have felt stiff when getting out of bed in the morning” in the last month. Each question was scored with a value of 1 for a positive answer, for a maximum score of 10. The second questionnaire was the Victorian Institute of Sport Assessment – Achilles (VISA-A), which is a reliable and valid measure of Achilles tendon pain [20]. The VISA-A provides a score out of 100 relative to the participant’s pain and function due to Achilles pain during activities that load the Achilles tendon. A score of 100 indicates no Achilles pain or functional impediment, while the lowest possible score of 0 indicates severely debilitating Achilles tendon pain.

### Achilles tendon structure

Ultrasound tissue characterisation (UTC) was used to provide a semi-quantifiable measure of the structure of both Achilles tendons, which has been validated against tendon biopsies in horses [21]. It also reveals reduced alignment of tendon fibrillar structure within two days of maximal exercise in Australian footballers [22], demonstrating that findings respond quickly to intervention. This was performed using previously described methods [11], where an ultrasound transducer (Smartprobe 10 L5; Terason 2000, Teratech, USA) is moved automatically by a tracker (UTC imaging, Kruisstraat, Netherlands) along the length of the tendon. In a standardised position of ankle dorsiflexion, hundreds of transverse images were taken and assembled using software (UTC 2010; UTC Imaging) to form a three-dimensional image [11].

To ensure adequate blinding, UTC files were assigned computer generated numbers separate from the participant identification numbers. The most painful tendon was then analysed, or the right tendon in those with no Achilles pain. A tendon volume was created, as previously decribed [11], from the disappearance of the calcaneus to the appearance of the triceps surae. This volume was then analysed by dedicated UTC software (UTC2010; UTC Imaging) and generated percentage compositions of the four different echo-types within the analysed tendon [11]. These echo-types represent tendon structure, where echotypes I and II denote overall aligned fibrillar structure and echotypes III and IV represent overall disorganised tendon structure (where I is slightly more favourable than II, and III more so than IV) [23, 24]. Mean total cross-sectional area (CSA) of the tendon was also measured, by calculating the average CSA based on the entire region of analysis (as described above). The mean normal CSA could then also be calculated using the percentage of echotypes I and II. Maximal anterior-posterior (AP) diameter was also measured using the UTC image.

### SNS measurement

Muscle sympathetic nerve activity (MSNA) was used as an in vivo measure of sympathetic drive, to document the effects of the active and placebo treatments on the SNS throughout the study to ensure a difference was obtained. As per previous protocols [11], participants fasted for 4–5 h before recordings, abstained from caffeine for 18 h and did not take regular medications (apart from the trial medication) on the morning of the recording.

Supine recordings of MSNA were made using a tungsten microelectrode (FHC, Bowdoinham, ME, USA) inserted directly into the peroneal nerve consistent with previously described methods [25, 26]. Heart rate (HR) (using electrocardiogram recordings) and beat-to-beat blood pressure (BP) were measured along with MSNA at rest over a 10-min period (PowerLab, model ML 785/8SP, ADI Instruments, NSW, Australia).

Analysis was performed on the Labchart program (version 5.5.5, ADI Instruments, Sydney, NSW, Australia) by manual, visual inspection of the MSNA neurogram, similar to previous work [11]. This was expressed as burst frequency (sympathetic bursts/min) and burst incidence (sympathetic bursts/100 heartbeats (HB)). Burst amplitude [27] was then used to calculate units per minute (bursts/min x mean burst amplitude) and units per 100HB (bursts/100HB × mean burst amplitude). This analysis was carried out by a sole investigator (EL), where intra-assessor variability is approximately 6% [11].

### Statistical analysis

As this was a secondary analysis, sample size was pre-determined by the original study [15] and limited by availability of resources in obtaining Achilles tendon measurements. Post-hoc power analysis was performed using G*Power 3.1 (Heinrich-Heine University) based on the difference between MSK scores for those receiving moxonidine. The power was calculated as 0.55 based on the effect size of 0.32 and the total sample size (*n* = 32). Group differences in continuous data were measured using independent t-tests for parametric data. 2-way ANOVA was used for multiple comparisons to determine if changes over time differed between the groups as a result of the different interventions, with adjustments for centred-baseline data and Bonferroni adjustments where appropriate (IBM SPSS Statistics version 22, IBM Corporation).

## Results

### Baseline characteristics

A total of 48 individuals were randomised in the original trial with 25 receiving placebo and 23 moxonidine [15]. 32 participants were recruited and followed up at the time MSK/Achilles tendon data was being collected after meeting the eligibility criteria (Fig. 1). 18 of these participants were in the placebo group, and 14 in the moxonidine group. Satisfactory UTC scans were obtained for 14/18 participants in the placebo group and 13/14 in the moxondine group. No side effects predominated in either group, with lack of energy and headache most commonly reported in each group [15]. The groups were equivalent for age and baseline metabolic data (Table 1), as well as hormonal profile (not shown). All data are reported as mean ± standard deviation unless otherwise stated.

**Table 1:** Baseline characteristics

	Placebo (*n* = 18)	Moxonidine (*n* = 14)	p-value (t-test)
Age	29.9 ± 5.9	30.8 ± 6.9	0.70
BMI	29.6 ± 5.1	30.9 ± 6.0	0.49
WHR	0.96 ± 0.05	0.96 ± 0.04	0.69
SBP	108 ± 11	115 ± 16	0.17
DBP	69 ± 9	72 ± 10	0.45
MAP	82 ± 9	86 ± 10	0.28
Fasting BGL	4.7 ± 0.5	4.7 ± 0.4	0.84
HOMA index	4.4 ± 2.4	4.0 ± 1.7	0.64
Matsuda index	4.0 ± 2.8	3.3 ± 2.5	0.48

*BMI* Body mass index, *WHR* Waist-to-hip circumference ratio, *SBP* Systolic blood pressure, *DBP* Diastolic blood pressure, *MAP* Mean arterial pressure, *BGL* blood glucose level, *HOMA* Homeostatic model assessment

### MSK and VISA-A scores

Sixteen women in the placebo group and ten in the moxonidine group had experienced some form of MSK pain at baseline in the last month. Analysis of the MSK questionnaires using 2-way ANOVA showed there was a small difference between placebo and moxonidine groups, but no difference between time-points (as a result of intervention) or when combing group and time-point (Table 2).

**Table 2:** MSK and VISA-A scores

Data	Group	Pre-intervention	Post-intervention	2-way ANOVA *p*-value
MSK score	Placebo	3.2 ± 2.2	2.8 ± 2.8	Group < 0.01* Time-point = 0.11 Group x Time-point = 0.69
	Moxonidine	2.1 ± 2.2	1.4 ± 2.1	
VISA-A	Placebo	88.2 ± 22.9	92.4 ± 13.5	Group = 0.01* Time-point = 0.29 Group x Time-point = 0.24
	Moxonidine	95.3 ± 6.6	95.1 ± 7.8	

*significant difference

Again, there was a difference between groups with regard to their baseline VISA-A scores. There were no significant differences in VISA-A score as a result of the intervention (Table 2). A ceiling effect was noted as only 4 participants in the placebo group had tendinopathy pre-intervention, and 3 in the placebo group and 1 in the moxonidine group post-intervention.

### UTC data

Satisfactory scans were obtained in 14 participants in the placebo group and 13 in the moxonidine group, with 4 scans in the placebo group and 1 in the moxonidine group of insufficient quality for analysis. Comparisons were made between percentage of favourable (I, II) and non-favourable (III and IV) echotypes, mean cross sectional area (CSA) (normal, pathological and total) and anterior-posterior diameter of the tendon (Table 3). Mean normal and total tendon CSA were slightly higher in the moxonidine group at baseline. There were no differences found as a result of the intervention.

**Table 3:** UTC data

Data	Group	Pre-intervention	Post-intervention	2-way ANOVA p-value
Echotype I + II %	Placebo	93.5 ± 5.6	96.0 ± 3.5	Group = 0.45 Time-point = 0.10 Group x Time-point = 0.64
	Moxonidine	94.9 ± 4.0	96.3 ± 3.2	
Mean normal CSA	Placebo	57.8 ± 12.6	59.6 ± 9.6	Group = 0.02* Time-point = 0.53 Group x Time-point = 0.92
	Moxonidine	65.9 ± 14.5	68.4 ± 14.3	
Mean total CSA	Placebo	61.9 ± 14.3	62.0 ± 9.9	Group = 0.03* Time-point = 0.81 Group x Time-point = 0.83
	Moxonidine	69.3 ± 15.0	71.0 ± 14.7	
AP diameter (mm)	Placebo	6.2 ± 1.1	6.1 ± 0.8	Group = 0.59 Time-point = 0.92 Group x Time-point = 0.51
	Moxonidine	6.2 ± 1.1	6.3 ± 1.2	

*significant difference

*CSA* Cross-sectional area, *AP* anterior-posterior

Note for placebo *n* = 14, moxonidine *n* = 13

### MSNA data

Basic cardiovascular measures, BP and HR, were obtained pre and post intervention for 17/18 participants in the placebo group (16 for HR) and 13/14 in the moxonidine group. All measures were no different between groups or as a result of intervention (see additional file 1).

Measurement of MSNA was affected by follow up, with only 14/18 participants from the placebo group and 9/14 from the moxonidine group obtaining appropriate measurements pre and post intervention for comparison. The moxonidine group had a lower SNS drive at baseline, however this was unaffected by the intervention in comparison to placebo (Table 4).

**Table 4:** MSNA data

Data	Group	Pre-intervention	Post-intervention	2-way ANOVA p-value
Burst frequency (bursts/min)	Placebo	31.7 ± 11.0	30.0 ± 16.7	Group = 0.03* Time-point = 0.34 Group x Time-point = 0.63
	Moxonidine	25.2 ± 7.9	20.0 ± 6.0	
Burst incidence (bursts/100HB)	Placebo	47.9 ± 13.1	46.3 ± 24.1	Group = 0.02* Time-point = 0.52 Group x Time-point = 0.76
	Moxonidine	37.4 ± 10.0	32.7 ± 10.1	
Units/min	Placebo	1624 ± 697	1477 ± 989	Group = 0.03* Time-point = 0.44 Group x Time-point = 0.93
	Moxonidine	1162 ± 338	977 ± 304	
Units/100HB	Placebo	2448 ± 877	2263 ± 1303	Group = 0.02* Time-point = 0.54 Group x Time-point = 0.96
	Moxonidine	1734 ± 429	1578 ± 460	

*significant difference

## Discussion

### Findings

In this study of women with PCOS, we examined the effect modulating the SNS had on MSK pain, Achilles pain and Achilles structure. It follows on from previous work which examined the involvement of muscle sympathetic nerve activity in people with Achilles tendinopathy, notably those with increased duration of symptoms [11].

We did not demonstrate a significant change in either group post intervention with regards to Achilles tendon or musculoskeletal symptoms. Given structural tendon changes are not always associated with pain and symptoms [28, 29], analysis of the effect moxonidine had on UTC data was of benefit in this population with few symptoms. Percentage of aligned fibrillar structure appeared to be similar to (if not slightly worse than) previous measures in elite Australian footballers [22], however tendon structure was not significantly changed by the 12-week intervention in either group. These findings suggest there is currently no evidence that blocking SNS activity with moxonidine for 12 weeks provides benefit for MSK or Achilles tendon pain, nor for Achilles tendon structure. This is of particular note in this population of women with PCOS, as this MSK pain may prevent women from undertaking physical activity as a vital part of their disease management [30–32].

### Limitations

This study has a number of key limitations that must be taken into consideration. The study has a small sample size due to it being a secondary analysis as part of a larger project investigating the effects of moxonidine in PCOS. The smaller sample size in comparison to the parent RCT was due purely to the premature end of access to resources for measuring MSK and Achilles data, so selection bias is minimised. However, this renders it underpowered in properly examining the effects of moxonidine on MSK and Achilles tendon pain, and definitive conclusions cannot be drawn from this paper.

Moreover, as recruitment in this study was based on a diagnosis of PCOS and not on Achilles tendon symptoms, a small number of participants began the study with pre-existing tendinopathy and the chances of significant change in either group was limited by this ceiling effect. Additionally, physical activity data were not measured, where differences among participants could plausibly affect tendon pain and structure measures.

Furthermore, the analysis of the MSNA data showed that while the moxonidine group had a lower sympathetic drive at baseline, there was no difference when comparing the effect of interventions between groups. Previous reports have shown that moxonidine is associated with reduced blood pressure and MSNA even in young normotensive subjects [33], yet systolic and diastolic blood pressure were also unaffected. It is possible that the intervention group were not compliant with their moxonidine dosages, although this did not seem to be a major issue as per the original RCT [15]. Moxonidine’s effect may also be changed in those with PCOS due to differences in adrenoreceptor expression [34], as one would expect moxonidine to reduce overall sympathetic drive and blood pressure. Regardless, the lack of significant change in sympathetic drive may provide another reason as to why the active intervention did not change tendon/MSK outcomes.

Therefore, while no changes in MSK or Achilles symptoms were found, it must be considered that this study was a secondary project to the original RCT and its role in the literature is predominantly descriptive and should guide future research in the area.

## Conclusion

This study of women with PCOS found no change in Achilles tendon pain, Achilles tendon structure or other MSK symptoms as a result of the sympatholytic medication. It was limited by its small sample size and inadequate modulation of SNS drive. While this study was the first to investigate the effect of blocking the SNS on MSK and Achilles tendon pain in a population with metabolic disease, there should be further studies of higher power, which recruit based on both tendon symptoms and metabolic disease, to investigate its true potential.

## Supplementary information

**Additional file 1.** BP and HR. Supplementary material: Blood pressure and heart rate. Measures of blood pressure and heart rate in both groups, before and after the intervention.

## Data Availability

The raw datasets generated and analysed during the current study are not publicly available, as this was not part of the participant consent process and a proportion of the data was obtained from the parent RCT, however they are available from the corresponding author on reasonable request.

## Abbreviations

MSK: Musculoskeletal
SNS: Sympathetic nervous system
T2DM: Type 2 diabetes mellitus
PCOS: Polycystic ovarian syndrome
RCT: Randomised controlled trial
BMI: Body mass index
WHR: Waist-to-hip circumference ratio
SBP: Systolic blood pressure
DBP: Diastolic blood pressure
MAP: Mean arterial pressure
HOMA: Homeostatic model assessment
VISA-A: Victorian Institute of Sport Assessment – Achilles
UTC: Ultrasound tissue characterisation
CSA: Cross-sectional area
AP: Anterior-posterior
MSNA: Muscle sympathetic nerve activity
HR: Heart rate
BP: Blood pressure
HB: Heartbeats
