# Supplementary material for: Does moxonidine reduce Achilles tendon or musculoskeletal pain in women with polycystic ovarian syndrome? A secondary analysis of a randomised controlled trial
Source: BMC Endocr Disord. 2020 Aug 26;20:131. doi: 10.1186/s12902-020-00610-8 (PMC7449016; doi:10.1186/s12902-020-00610-8)
Supplement: Supplementary file 1 — Additional file 1. BP and HR. Supplementary material: Blood pressure and heart rate. Measures of blood pressure and heart rate in both groups, before and after the intervention. [file 12902_2020_610_MOESM1_ESM.docx]

Supplementary material: Blood pressure and heart rate.

| Data | Group | Pre-intervention | Post-intervention | 2-way ANOVA p-value |
| --- | --- | --- | --- | --- |
| SBP | Placebo | 107 ± 9.6 | 108 ± 12.1 | Group = 0.16  Time-point = 0.76  Group x Time-point = 0.43 |
|  | Moxonidine | 114 ± 16.2 | 110 ± 11.0 |  |
| DBP | Placebo | 68.4 ± 9.1 | 68.0 ± 7.5 | Group = 0.47  Time-point = 0.65  Group x Time-point = 0.79 |
|  | Moxonidine | 70.5 ± 9.4 | 69.0 ± 6.3 |  |
| MAP | Placebo | 81.2 ± 8.8 | 81.5 ± 8.2 | Group = 0.27  Time-point = 0.67  Group x Time-point = 0.59 |
|  | Moxonidine | 85.0 ± 10.5 | 82.8 ± 7.2 |  |
| HR | Placebo | 64.1 ± 8.5^^^ | 62.6 ± 8.4^^^ | Group = 0.46  Time-point = 0.11  Group x Time-point = 0.33 |
|  | Moxonidine | 68.3 ± 10.8 | 62.0 ± 9.1 |  |

Note for placebo n = 17 (^n = 16), moxonidine n = 13
